# Modeling consolidation of soft clay by developing a fractional differential constitutive model in conjunction with an intelligent displacement inversion method

**DOI:** 10.1371/journal.pone.0275034

**Published:** 2022-09-30

**Authors:** Zhen Liu, Wei Hu, Weihua Ming, Shenghua Xiong, Cuiying Zhou, Lihai Zhang

**Affiliations:** 1 Sun Yat-sen University, Guangzhou, China; 2 Guangdong Engineering Research Centre for Major Infrastructure Safety, Sun Yat-sen University, Guangzhou, China; 3 Department of Infrastructure Engineering, The University of Melbourne, Melbourne, Australia; NIT Patna: National Institute of Technology Patna, INDIA

## Abstract

Studying the constitutive relation of soft clays is of critical importance for fundamentally understanding their complex consolidation behavior. This study proposes a fractional differential constitutive model in conjunction with an intelligent displacement inversion method based on the classic particle swarm optimization for modeling the deformation behavior of soft clay. The model considered the rheological properties of soft clay at different consolidation stages. In addition, statistical adaptive dynamic particle swarm optimization-least squares support vector machines were implemented to identify the model parameters efficiently. The accuracy and effectiveness of the model were validated using available experimental results. Finally, the application results showed that the proposed model could efficiently simulate coupling properties of soft clay’s primary and secondary consolidations.

## 1. Introduction

Soft clays are widely distributed in coastal regions [1]. For construction projects built on soft foundations, the large settlement problem is quite prominent and may even cause damage to structures [2]. Therefore, it is critical for geotechnical engineers to fundamentally understand the constitutive relationship of soft clay and identify the critical factors that govern the settlement of soft foundations.

With the advancement of computing power, the implementation of large-scale numerical simulations to solve complex settlement problems of soft foundations has become increasingly popular [3]. However, the accuracy of the simulation outcome is highly dependent on the parameters that describe the mechanical properties of soft clay [4]. In particular, the nonlinear rheological properties of soft clay present a further challenge in numerical modeling [5, 6]. The current modeling parameter estimation is generally based on intelligent displacement back analysis involving an iterative procedure, which may lead to simulations of low efficiency [7]. Therefore, further development of the theoretical model is necessary.

Fractional calculus, a mathematical tool for solving nonlinear models in complex physical and mechanical systems, has been implemented in viscoelastic and viscoplastic models [8–10]. Owing to its high degree of computational efficiency, fractional calculus has been used to study the constitutive relationship between complex rock and soil materials. Zhou et al. proposed a new creep constitutive model based on time-based fractional derivatives using the fractional derivative Abel dashpot [11], whereas Yin et al. used fractional orders to quantitatively describe the elasticity and viscosity of geotechnical materials [12]. Zhu et al. presented a preliminary study to model the one-dimensional compression of soft clay using fractional derivatives [13]. However, the present fractional calculus model was mainly implemented by assuming linear instantaneous deformation without considering the coupling characteristics of the entire stage of primary and secondary consolidations of soft clay.

To improve the accuracy of parameter acquisition and simulation efficiency, intelligent algorithms, such as genetic algorithms, evolutionary algorithms, and ant colony algorithms, have been gradually introduced in the field of displacement back analysis [14, 15]. Some artificial intelligence methods, such as neural networks, are also applicated in the parameters back analysis of soft clay [16, 17].

Particle swarm optimization (PSO), which is a swarm intelligence optimization algorithm, has been widely used in rock parameter acquisition owing to its simple implementation and high computational efficiency [18]. For example, the hybrid moving boundary PSO algorithm was used to identify model parameters of the field pressure gauge test [19]. Zhao and Yin used PSO to improve the generalization performance of the search process in the support vector machine (SVM) model aiming to increase the efficiency and precision of the back analysis of geomechanical parameters [20]. In addition, hybrid genetic programming with a modified PSO algorithm has been proposed to investigate the viscoelastic behavior of rocks [21]. However, owing to the high degree of complexity of soft clay, the classic PSO algorithm encounters difficulties while converging [19, 22, 23]. Therefore, it is necessary to develop a new soft clay fractional differential constitutive model to optimize the intelligent displacement inversion algorithm of the model parameters.

In the present study, the coupling of primary and secondary consolidations was established using a fractional differential element, instantaneous yield surface, and viscous yield surface. Theoretically, a new fractional differential constitutive model for soft clay was developed. In addition, an algorithm using statistical adaptive dynamic particle swarm optimization (SADPSO) was presented to solve the issues of unreasonable inertia alterative method and local extremum of the traditional PSO. Moreover, an algorithm for statistical adaptive dynamic particle swarm optimization-least squares support vector machines (SADPSO-LSSVM) was developed to improve computational efficiency. Practically, the reliability of the model was verified by comparing the experimental results with the model’s predictions. The expressway project application showed that the theory has a significant value. The fractional differential constitutive model can be applied to settlement calculation and verified using the finite element method (FEM).

## 2. Methods

### 2.1 Fractional differential constitutive model for soft clay

The model was established by considering characteristics of the consolidation deformation of soft clay as follows

- The total strain in soft clay consists of instantaneous elastic strain, instantaneous plastic strain, viscoelastic strain, and viscoplastic strain.
- The rheological characteristics of soft clay can be described as the coupling deformations of primary and secondary consolidations [24–26].
- The instantaneous and viscous deformations of soft clay are generally nonlinear [27].

In the rheological theory of soft clay, an element model can be formed to simulate the stress–strain–time relationship by connecting various elements in series or in parallel. To solve the problems of nonlinear rheological behavior of soft clay and the combination of primary consolidation deformation and secondary consolidation deformation, a fractional differential constitutive model of soft clay is proposed using the fractional differential theory and classic theoretical model of viscoelastic–plastic components. The viscous yield surface replaces the Saint-Venant body of the element model [28].

**Fig 1:**
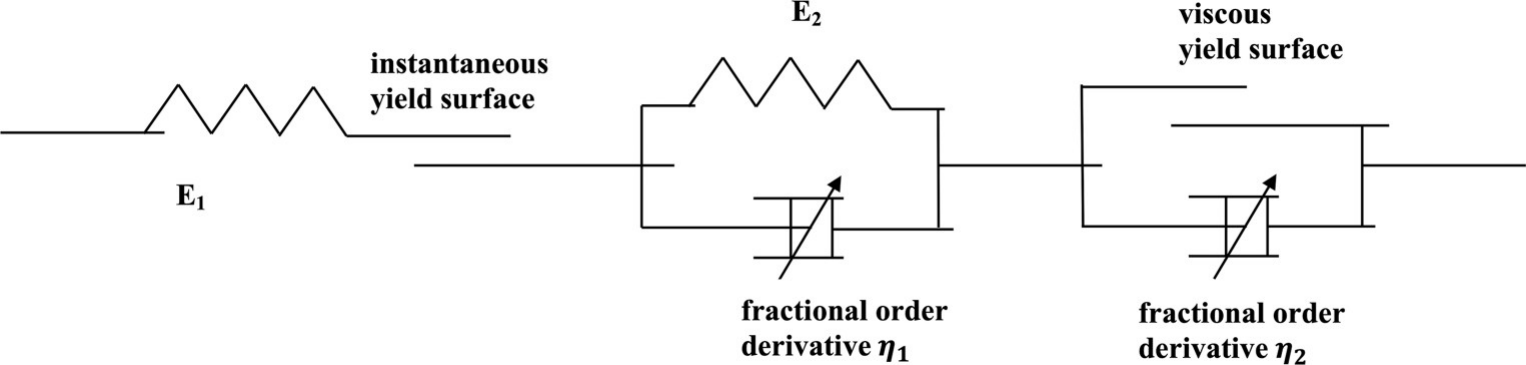
Developed fractional differential constitutive model of soft clay.

Thus, the total strain relation in the constitutive relation of soft clay can be expressed as

$$\varepsilon =\varepsilon_{ie}+\varepsilon_{ip}+\varepsilon_{ve}+\varepsilon_{vp},$$ (1)

where *ε*_*ie*_ is the instantaneous elastic strain represented by the Hooker’s body, *ε*_*ip*_ is the instantaneous plastic strain represented by the instantaneous yield surface, *ε*_*ve*_ is the viscoelastic strain represented by the combination of the Hooker’s body and fractional differential element, and *ε*_*vp*_ is the viscoplastic strain represented by the combination of the viscous yield surface and fractional differential element. The fractional differential constitutive model of soft clay is described in Fig 1.

The stress–strain relationship of the instantaneous elastic deformation can be expressed by Eq (2) as follows:

$$\varepsilon_{ie}=\frac{1}{E_{1}}\sigma ,$$ (2)

Where *E*_1_ is the elastic modulus and *σ* is the stress.

**Fig 2:**
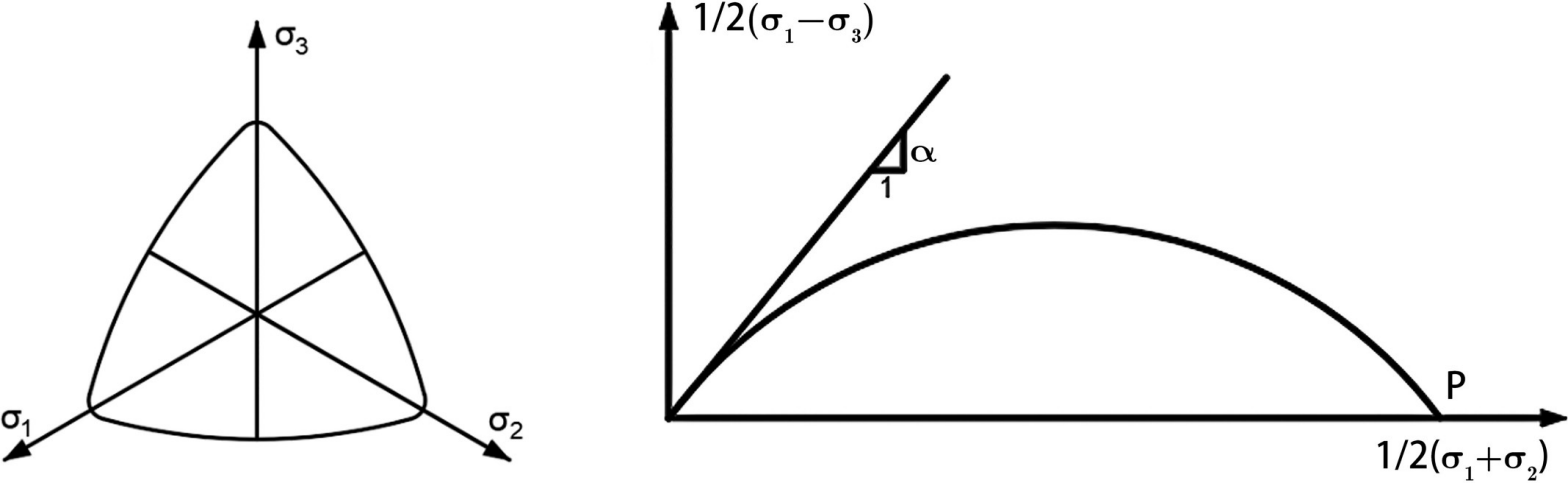
Instantaneous yield surface.

To describe the instantaneous plastic strain of the fractional differential constitutive model of soft clay and the instantaneous yield surface in the instantaneous plastic body, the double hardening yield equation was used [29]. That is,

$$f_{s}=\frac{\sigma_{m}}{1-(\frac{\eta }{\alpha }{)}^{n}}-p,$$ (3)

where $\sigma_{m}=\frac{1}{3}(\sigma_{1}+\sigma_{2}+\sigma_{3})$; $\eta =\frac{1}{\sqrt{2}}[(\frac{\sigma_{1}-\sigma_{3}}{\sigma_{1}+\sigma_{3}}{)}^{2}+(\frac{\sigma_{1}-\sigma_{2}}{\sigma_{1}+\sigma_{2}}{)}^{2}+(\frac{\sigma_{2}-\sigma_{3}}{\sigma_{2}+\sigma_{3}}{)}^{2}{]}^{1/2}$; *p* and *α* are hardening functions of the instantaneous plastic body strain $\varepsilon_{v}^{p}$ and instantaneous plastic shear strain $\varepsilon_{s}^{p}$, respectively; $p=p_{0}\;\exp(\frac{\varepsilon_{v}^{p}}{c_{c}-c_{s}})$; $\alpha =\alpha_{m}-(\alpha_{m}-\alpha_{0})\exp(-\frac{\varepsilon_{s}^{p}}{c_{\alpha }})$ [29]. The critical state constant *M* is obtained by the parameter *α*, and the the shape of the yield surface in the *π* and *z* planes asis shown in Fig 2.

The incremental equation for the instantaneous plastic strain can be obtained using Eq (3) as follows:

$$\{\Delta \varepsilon_{ip}\}=\frac{1}{H_{s}}\{\frac{\partial f_{s}}{\partial \sigma }\}\{\frac{\partial f_{s}}{\partial \sigma }{\}}^{T}\{\Delta \sigma \},$$ (4)

where, *H*_*s*_ is the hardening modulus [29].

The viscoelastic component is composed of a spring element and fractional differential element connected in parallel. The stress–strain relationship that describes the elastic hysteresis of the soil can be obtained as follows:

$$\sigma =E_{2}\varepsilon_{ve}+\eta_{1}D^{q_{1}}\varepsilon_{ve},$$ (5)

where *E*_2_ denotes the elastic modulus of the spring element, *η*_1_ denotes the viscosity coefficient of the fractional differential element, *q*_1_ denotes the differential order of the fractional differential element (0<*q*_1_<1), and *D* denotes the fractional differential operator. The viscoplastic strain can be obtained using Eq (5) as follows:

$$\varepsilon_{ve}=J_{ve}(t)\sigma ,$$ (6)

where *J*_*ve*_ is the creep compliance of viscoelastic strain which can be expressed as

$$J_{ve}(t)=\frac{1}{E_{2}}[-\sum_{k=1}^{\infty }(-1{)}^{k}(\frac{\eta_{1}}{E_{2}}{)}^{-q_{1}k}\frac{t^{q_{1}k}}{\Gamma (1+q_{1}k)}].$$ (7)

Viscoplastic strain can be described using the Bingham model [30], in which the viscous element is a Newtonian body and the plastic elements are Saint-Venant bodies. To describe the viscoplastic strain of soil more effectively, the Bingham model is further improved in this study by (a) replacing a Newtonian body with a fractional differential element and (b) replacing the Saint-Venant body with the slide described by the viscous yield surface. By changing the stress over time, the viscous yield surface equation can be obtained as follows:

$$f_{d}=\frac{\sigma_{m}}{1-(\frac{\eta }{\alpha_{t}}{)}^{n}}-p_{t}.$$ (8)

The hardening functions of the viscous yield surface equation can be defined as follows:

$$p_{t}=p_{0}\;\exp(\frac{\varepsilon_{v}^{vp}}{c_{c}-c_{s}}),$$ (9)

$$\alpha_{t}=\alpha_{m}-(\alpha_{m}-\alpha_{0})\;\exp(-\frac{\varepsilon_{s}^{vp}}{c_{\alpha }}),$$ (10)

where $\varepsilon_{v}^{vp}$ the viscoplastic volumetric strain, and $\varepsilon_{s}^{vp}$ is the viscoplastic shear strain. Based on the improved Bingham model, the stress–strain relationship of the viscoplastic part can be described as

$$\sigma =\eta_{2}D^{q_{2}}\varepsilon_{vp}+\sigma_{2},$$ (11)

where *σ*_2_ is the plastic stress, and $\eta_{2}D^{q_{2}}\varepsilon_{vp}$ is the viscous stress of the fractional differential elements. Considering a one-dimensional problem, Eq (11) can be rewritten as $D^{q_{2}}\varepsilon_{vp}=\frac{1}{\eta_{2}}<(\sigma -\sigma_{2})>$. <(*σ*−*σ*_2_)>, which is a switch function. For the three-dimensional case, the coincidence of the plastic potential surface and yield surface leads to

$$D^{q_{2}}\varepsilon_{vp}=\frac{1}{\eta_{2}}<\Phi (f_{d})>\left\{\frac{\partial f_{d}}{\partial \sigma }\right\},$$ (12)

where *f*_*d*_ is a function of viscous yield surface, and <*Φ*(*f*_*d*_)> is the switch function, which can be defined as

$$<\Phi (f_{d})>=\left\{ \begin{array}{c} 0,\;\;\;\;\;f_{d}\leq 0,\\ \Phi (f_{d}),\;\;\;f_{d}>0,f_{s}>0. \end{array}\right.$$ (13)

As $<\Phi (f_{d})>\{\frac{\partial f_{d}}{\partial \sigma }\}$ is time-dependent, by letting $Q(t)=<\Phi (f_{d})>\{\frac{\partial f_{d}}{\partial \sigma }\}$, we obtain

$$D^{q_{2}}\varepsilon_{vp}=\frac{1}{\eta_{2}}\sum_{k=0}^{+\infty }\frac{Q^{\left(k\right)}(0)}{k!}t^{k}.$$ (14)

Assuming *Q*^(*k*)^(*t*) is a k-order derivative equaling *Q*^(*k*)^(0) when t = 0, the Laplace transformation of Eq (14) leads to

$${\overset{¯}{\varepsilon }}_{vp}=\frac{1}{\eta_{2}}\sum_{k=0}^{+\infty }Q^{\left(k\right)}(0)p^{-(q_{2}+k+1)},$$ (15)

where $\overset{¯}{\varepsilon }(p)$ denotes the Laplace transformation of *ε*(*t*). By performing the inverse Laplacian transformation on both sides of Eq (15) and using $L^{-1}[p^{-r}]=\frac{t^{r-1}}{\Gamma (r)}$, we obtain

$$\varepsilon_{vp}=t^{q_{2}}/\eta_{2}\sum_{k=0}^{+\infty }Q^{\left(k\right)}(0)t^{k}/\Gamma (q_{2}+k+1).$$ (16)

Ignoring the higher order derivative term of the order k leads to,

$$\varepsilon_{vp}=\frac{t^{q_{2}}}{\eta_{2}}\frac{1}{\Gamma (q_{2})(2q_{2}^{2}+q_{2})}<\Phi (f_{d})>\left\{\frac{\partial f_{d}}{\partial \sigma }\right\}.$$ (17)

Based on the switch function of the viscoplastic strain proposed in previous studies [31], we obtain

$$\Phi (f_{d})=\frac{f_{d}}{f_{0}},$$ (18)

where *f*_*d*_ is the function of the viscous yield surface, and *f*_0_ is a dimensionless coefficient (*f*_0_ = 1 in this study).

The stress-strain relationship of the fractional differential constitutive model can be described using Eqs (1), (2), (4), (6), and (17). The parameters of the constitutive model (11 parameters in total) include elastic modulus *E*_1_, viscoelastic parameters(*E*_2_, *η*_1_, *q*_1_), plastic parameters (*λ*, *κ*, *e*_0_, *φ*_*r*_, *p*_*c*_) and viscoplastic parameters (*η*_2_, *q*_2_). Although the plastic parameters (*λ*, *κ*, *e*_0_, *φ*_*r*_, *p*_*c*_) can be determined by conventional triaxial compression tests, the other model parameters can be estimated through the calibration process using the intelligent displacement inversion method.

### 2.2 Intelligent displacement inversion method

The intelligent displacement inversion method used in this study involves establishing an objective function based on a known displacement sequence. The optimization process was carried out using SADPSO-LSSVM, which can efficiently solve the complex optimization problem. The objective function was developed based on previous studies [32]. That is,

$$F(P)=\sum_{i=1}^{q}\sum_{j=1}^{k}(FEM_{ij}(P)-u_{ij}{)}^{2},$$ (19)

where (*P*) is the set of parameters to be inverted. *FEM*_*ij*_(*P*) and *u*_*ij*_ are the numerical and measured values of the displacement *j* component at the *i-*th measurement point, respectively. *q* is the number of displacement measurement points, and *k* is the number of displacement components.

#### 2.2.1 Statistical adaptive dynamic particle swarm optimization

PSO is a stochastic optimization algorithm constructed by simulating the swarm behavior of birds [33]. Through optimizing the local and global search abilities, an improved particle swarm optimization algorithm with inertia weight called “standard particle swarm optimization algorithm” was adopted [34]. To derive the algorithm, the inertia weight must first be defined. The inertia weight *w*_*i*_ can be defined as [35],

$$w_{i}=w_{max}-(w_{max}-w_{min})\frac{t}{N},$$ (20)

where *w*_*max*_ and *w*_*min*_ are the maximum and minimum values of the inertia weight, respectively. *t* is the current number of iterations, and *N* is the maximum number of iterations.

Eq 20 shows that inertia weight decreases linearly with the number of iterations. Therefore, the equations for the standard particle swarm algorithm are:

$$\left\{ \begin{array}{c} v_{ij}(t+1)=w_{i}v_{ij}(t)+c_{1}r_{1j}(t)(p_{ij}(t)-x_{ij}(t))+c_{2}r_{2j}(t)(p_{gj}(t)-x_{ij}(t)),\\ x_{ij}(t+1)=x_{ij}(t)+v_{ij}(t+1), \end{array}\right.$$ (21)

where *c*_1_ and *c*_2_ are the cognitive constant and social constant, respectively, *r*_1_ and *r*_2_ are random numbers that are uniformly distributed in the range of 0–1. The *i*-th particle is denoted as *P*_*i*_ = (*p*_*i*1_, *p*_*i*2_,…,*p*_*ij*_)^*T*^, and *g* is the index of the particle in the swarm.

However, the inversion method based on PSO has some limitations. The global optimal particle may not be updated effectively in the optimization process, and the linear decrease in the inertia weight may compromise the local search ability of the algorithm at the initial stage of the iteration. To overcome the above challenges in modeling soft clay, the following improvements were made:

1. *Define the dynamic neighborhood structure of particles according to their spatial positions*
  In each iteration, the distances between the particles are calculated, and a particle group is determined based on the maximum distance between two particles (*dis*_*max*). Based on the critical distance proposed by Suganthan [36], the neighborhood of a particle group is gradually extended to include all particles as the number of iterations increases. Thus, the Eq (21) can be improved as
$$v_{ij}(t+1)=w_{i}v_{ij}(t)+c_{1}r_{1j}(t)\left(p_{ij}(t)-x_{ij}(t)\right)+c_{2}r_{2j}(t)\left(p_{lj}(t)-x_{ij}(t)\right),$$ (22)
  where *p*_*lj*_(*t*) represents the optimal location of the particle neighborhood.
2. *Improvement of adaptive inertia weight*
  As linear decrease in the inertia weight leads to limited global searching ability in the particle swarm, the adaptive evolution equation of the inertia weight is obtained by further developing Eq (20) as
$$w_{i}=w_{max}-(w_{max}-w_{min})\frac{t}{N}R_{i},$$ (23)
  where *R*_*i*_ represents adaptive function. That is,
$$R_{i}=1-\frac{1+d_{i}}{1+d_{max}}\frac{f_{i}-f_{min}+1}{f_{max}-f_{min}+1},$$ (24)
  where *d*_*i*_ is the distance between the particle and global optimal particle, *d*_*max*_ is the longest distance from the global optimal particle, *f*_*i*_ is the adaptive value of the particle, and *f*_*min*_ and *f*_*max*_ are the minimum and maximum adaptive values of the particles, respectively. $\frac{1+d_{i}}{1+d_{max}}$ describes the relationship between the inertia weight and distance between the particle and global optimal position. A smaller value of the inertia weight represents a shorter distance between the particle and global optimal position. In addition, $\frac{f_{i}-f_{min}+1}{f_{max}-f_{min}+1}$ describes the relationship between the inertial weight and adaptive value of the particle. A smaller adaptive value represents a better local convergence ability. The dynamic adaptive process of the inertia weight can improve the global search ability of the particle swarm.
3. *Accelerating step evolution based on statistical analysis*

In the PSO process, the identification of the optimal step size is challenging. Therefore, an accelerating step size was developed based on a statistical analysis of the model parameters, such as *c*_*1*_ and *c*_*2*_. The acceleration step size is defined based on the particle adaptive value *f*_*i*_. The details are as follows:

- When *f*_*i*_−*μ*<−*σ*, the adaptive value *f*_*i*_ is relatively small, and the particle is close to the target of optimization. To improve the convergence process, the step size to its optimal position should be large, whereas the step size to the global optimal position should be small. The acceleration step can be defined as
$$\left\{ \begin{array}{c} c_{i1}=c_{1max},\\ c_{i2}=c_{2min}, \end{array}\right.$$ (25)
  where *c*_*i*1_ is the step size of the *i-*th particle towards its optimal position, and *c*_*i*2_ is the step size of the *i-*th particle towards the global optimal position.
- When *f*_*i*_−*μ*>*σ*, the adaptive value *f*_*i*_ is relatively large, and the particle is far from the optimization target. To improve the convergence process, the step size to its optimal position should be small, whereas the step size to the global optimal position should be large. The acceleration step can be defined as
$$\left\{ \begin{array}{c} c_{i1}=c_{1min},\\ c_{i2}=c_{2max}, \end{array}\right.$$ (26)
- When |*f*_*i*_−*μ*|≤*σ*, the adaptive value *f*_*i*_ is relatively moderate, and the distance between the particle and its target of optimization is relatively moderate. The dynamic adaptive acceleration step can be expressed as
$$\left\{ \begin{array}{c} c_{i1}=c_{1max}-(c_{1max}-c_{1min})\times \frac{t}{N}\times R_{i},\\ c_{i2}=c_{2max}-(c_{2max}-c_{2min})\times \frac{t}{N}\times R_{i}, \end{array}\right.$$ (27)

To validate the SADPSO algorithm, its results are compared with those from the following two test functions of the classic PSO algorithm [36]:

$$f_{0}(x)=\sum_{j=1}^{n}x_{j}^{2},$$ (28)

$$and\;f_{1}(x)=\sum_{j=1}^{n}[x_{j}^{2}-10\;\cos(2\pi x_{j})+10].$$ (29)

Each test function considers cases with 20, 30, and 50 dimensions. Forty particles were involved in this study with a maximum evolution algebra of 1000, an initial inertia weight of 0.95, and a final inertia weight of 0.2. The maximum and minimum values of *c*_1_ and *c*_2_ are 2 and 0.2, respectively. The determination distance of the dynamic neighborhood is defined as *Frac* = (3*t+0.6*N)/*N*, where *t* is the number of iteration steps, and *N* is the total number of iteration steps. The selection of the dynamic neighborhood of a particle is based on the fact that the ratio of *dis*_*ij*_ to *dis*_*max* should be greater than the critical determination distance *Frac* [36]. Each test function was tested for 50 times, and the results were presented in Table 1.

**Table 1:** Comparison of classic particle swarm optimization with statistical adaptive dynamic particle swarm optimization results.

Function	Dimension	Classic PSO		SADPSO		
		Maximum error	Total error	Maximum error	Total error	Error reduction
*f*_0_(*x*)	20	0.000000	0.000000	0.000	0.0	0.0%
	30	0.000006	0.000043	0.000	0.0	100.0%
	50	0.892900	9.162000	0.027	0.1	98.9%
*f*_1_(*x*)	20	51.74	1516.10	46.77	1208.10	20.3%
	30	106.59	3506.85	86.57	2559.40	27.0%
	50	383.00	11745.30	205.81	6672.20	43.2%

From the results of function calculations, the error of SADPSO has a significant reduction compared to the classic PSO, especially in the high-dimensional case. According to the applied function, the SADPSO algorithm is suitable for optimizing a problem with a relatively small sample size without special computational efficiency requirements.

#### 2.2.2 Adaptive dynamic particle swarm optimization- least squares support vector machines

1. The process of algorithm implementation
  The least-squares support vector machine (LSSVM) algorithm is an improved support vector machine based on statistical learning theory [37]. The algorithm in regression analysis using nonlinear feature mapping leads to
$$\Phi :\;R^{m}\to H,$$ (30)
  where *H* denotes the feature space. By transforming the nonlinear regression in the original input space using an optimal linear regression function through data fitting, we obtain
$$y(x)=w^{T}\Phi (x)+b,$$ (31)
  where *w* is the weight vector in the space *H*, and *b*∈*R* is the offset vector.
  By replacing the inner product operation of the linear regression in the original input space with the kernel function, we obtain
$$K(x_{k},x_{l})=\Phi^{T}(x_{k})\Phi (x_{l}).$$ (32)
  Using a structured risk function, the regression analysis problem can be transformed into a risk minimization problem. That is,
$$\min J\;(w,e)=(w^{T}\cdot w)/2+C(\sum_{i=1}^{n}{e_{k}}^{2})/2,\;st.\;y_{k}=w^{T}\Phi (x_{k})+b+e_{k},$$ (33)
  where *C* is an adjustable regularization parameter, and *e*_*k*_ is an error variable. The dual theory is introduced to establish the Lagrange equation to solve the optimization problem [37]. That is,
$$L(w,b,e,\alpha )=\frac{1}{2}w^{T}\cdot w+\frac{1}{2}C(\sum_{i=1}^{n}{e_{k}}^{2})-\sum_{i=1}^{n}\alpha_{i}[w^{T}\Phi (x_{i})+b+e_{i}-y_{i}],$$ (34)
  where *α*_*i*_ is the Lagrange multiplier. According to the optimization conditions $\frac{\partial L}{\partial w}=0,\frac{\partial L}{\partial \alpha }=0,\frac{\partial L}{\partial b}=0,\;and\;\frac{\partial L}{\partial e}=0$, the weight vector *w* can be obtained. Meanwhile, the kernel function *K*(*x*_*k*_, *x*_*l*_) is used to replace *Φ*^*T*^(*x*_*k*_)*Φ*(*x*_*l*_) so that the optimization problem can be described by a series of linear equations. That is,
[01⋯11K(x1,x1)+1C⋯K(x1,xn)⋮⋮⋱⋮1K(xn,x1)⋯K(xn,xn)+1C] [bα1⋮αn]=[0y1⋮yn]. (35)
2. The step to solve parameters
  As the convergence accuracy of the algorithm is significantly affected by the kernel parameters *δ* and regularization parameter *C*, it is necessary to optimize these two parameters of the LSSVM. Owing to its better global searching ability and simple implementation, the SADPSO algorithm is used in the parameter optimization of the LSSVM (i.e., SADPSO–LSSVM).

The regularization parameter *C* and kernel parameter *δ* were first optimized using SADPSO-LSSVM. In the LSSVM algorithm, the kernel function generally selects the radial basis function, that is,

$$K(x_{k},x_{l})=\exp(-||x_{k}-x_{l}||/2\delta^{2}),$$ (36)

where *δ* is the kernel parameter. The learning and test sample sets were obtained using a sample time series set. The k-fold cross validation was then used to further optimize the parameters. The LSSVM model was established using the optimized parameters *δ* and *C*, and the corresponding regression analysis was performed.

Statistical adaptive dynamic particle swarm optimization is an improved particle swarm algorithm, which mainly implements three improvements: the dynamic neighborhood velocity evolution equation, the adaptive inertia weight evolution equation, and the statistical acceleration step evolution equation. The specific steps of optimizing the regularization parameter *C* and the kernel parameter *δ* of the LSSVM algorithm based on the statistical adaptive dynamic neighborhood particle swarm algorithm are as follows:

1. Set the initial parameters of the statistical adaptive dynamic neighborhood particle swarm algorithm, including the group size s, the maximum and minimum values of acceleration c1, c2, the maximum and minimum values of inertia factor, the maximum number of iterations, and the iteration stop criterion, etc.;
2. Determine the value range of the regularization parameter *C* and the kernel parameter *δ* of the LSSVM algorithm;
3. The random position and velocity of the particle swarm are set based on the initialization process. And the random position of the particle is taken as a set of values of the regularization parameter *C* and the kernel parameter *δ*;
4. Define the mean squared error function of k-fold cross validation as the fitness function;
5. The judging distance that defines the dynamic neighborhood is *Frac* = (3*t+0.6*N)/*N*, where t represents the number of iteration steps, and N represents the total number of iteration steps.
6. The dynamic neighborhood range of each particle is determined by the selection basis of particle dynamic neighborhood;
7. Calculate the fitness value of each particle in the group according to the fitness function, and change the historical optimal position of the particle and the local optimal position of the particle neighborhood at the same time. The steps are:
  - The fitness value of each particle is compared with the fitness value of the historical optimal position, and if it is better, its position is set as the historical optimal position of the particle.
  - The fitness value of each particle is compared with the fitness value of the optimal position experienced by the particle’s dynamic neighborhood. If it is better, its position is set as the local optimal position of the particle neighborhood.
8. Adjust the parameters of each particle.
  - Change the inertia weight of each particle according to the adaptive inertia weight evolution Eqs (23) and (24);
  - Change the acceleration step size of each particle according to the statistical acceleration step size evolution Eqs (25), (26) and (27);
  - Change the current velocity of each particle according to the velocity evolution Eq (22) of the dynamic neighborhood;
  - Change the current position of each particle according to the position evolution Eq (21);
9. Determine whether the fitness value satisfies the iterative stop criterion or whether it reaches the maximum evolutionary number. If not, return to step (6) to recalculate; otherwise, end the process and get the required result.

#### 2.2.3 Intelligent displacement inversion algorithm for soft clay

**Fig 3:**
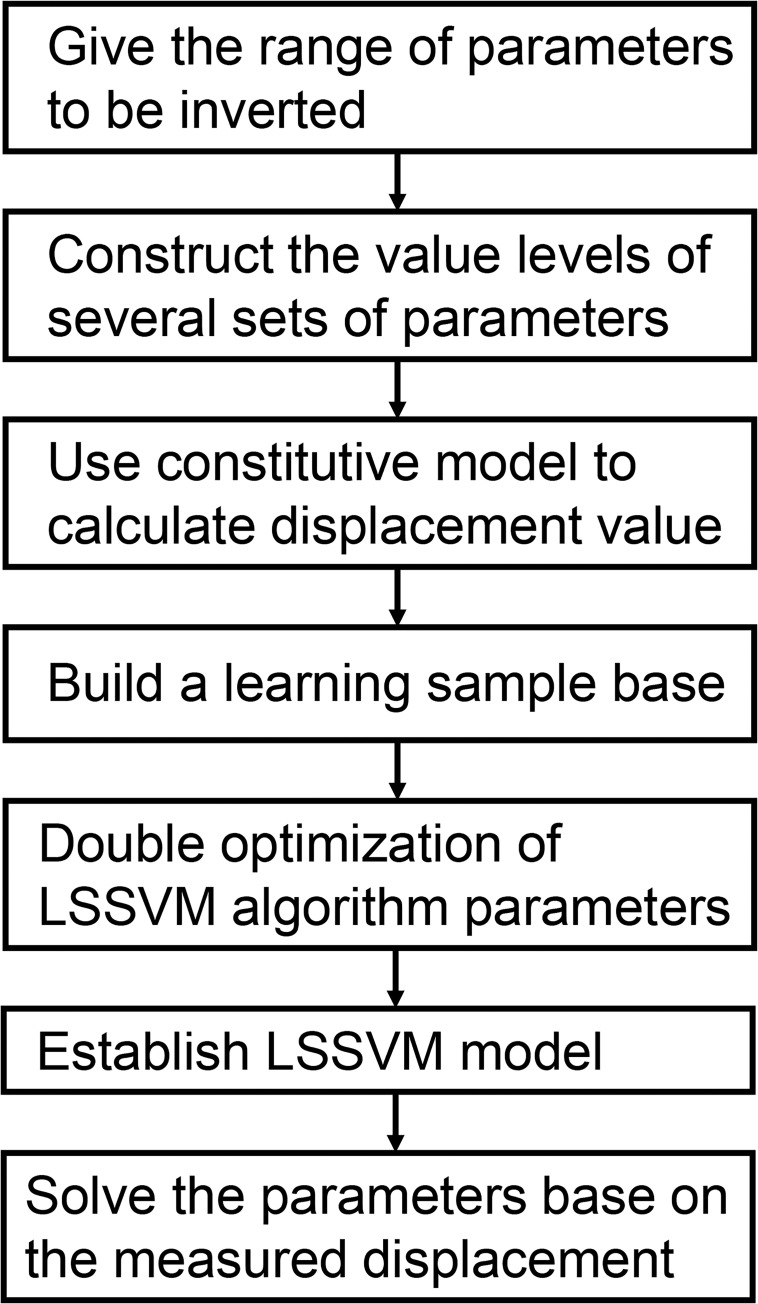
The procedure of implementing the intelligent displacement inversion algorithm in the constitutive model.

Fig 3 shows the procedure for implementing the intelligent displacement inversion algorithm in the constitutive model to identify the optimal global model parameters.

The detailed implementation steps for the intelligent displacement algorithm are as follows:

1. Define the search ranges of each parameter to be inverted, such as *E*_1_, *E*_2_, *η*_1_, *q*_1_, *η*_2_ and *q*_2_ (they are defined based on practical engineering experience).
2. Divide the search ranges of each parameter further into several sublevels. Then, combine the parameters with different sublevels into several groups based on the orthogonal experiment design.
3. Calculate the displacement of each measurement point using the developed fractional differential constitutive model based on the test combinations.
4. Establish a learning sample database based on the displacement and spatial location of each measurement point.
5. Use the SADPSO algorithm and k-fold cross validation for model parameter optimization using the LSSVM algorithm.
6. Establish the LSSVM model based on the optimized parameters. The specific steps are as follows:
  - The learning sample base calculated by the fractional differential constitutive model is used as the training sample set for the LSSVM.
  - The kernel function is defined as the inner product form of the high-dimensional feature space, *i*.*e*., the kernel function is $K(x_{k},x_{l})=\exp(-||x_{k}-x_{l}||/2\delta^{2})$, where *δ* is kernel parameter.
  - The kernel matrix is constructed using the kernel parameter values obtained by double optimization and the regularized training sample set, which is in the range of -1–1.
  - Based on the regularization parameter *C* obtained by double optimization, the Lagrange multiplier *α*_*i*_ and hyperplane coefficient *b* are obtained by solving Eq (35) using the least-squares method.
  - The output mapping function $f(x)=\sum_{i=1}^{n}\alpha_{i}K(x,x_{i})+b$ is established.
  - The kernel matrix *K*(*x*, *x*_*i*_), based on the predicted value *x*, is established.
7. Define the critical distance of the dynamic neighborhood as *Frac* = (3*t+0.6*N)/*N*, where *t* represents the number of iterative steps, and *N* represents the total number of iterations. Based on the initialization process, several groups of parameters to be inverted were randomly generated. The corresponding displacement value of each particle was calculated using the LSSVM model, and the objective function was used for optimization. Based on the SADPSO algorithm, an evolutionary search was performed until the optimal parameter values were obtained.

## 3. Testing results and discussion

In this section, the model predictions are compared with the triaxial creep testing results of grey soft clay in Shanghai, China [38]. Specifically, the axial strains of soil samples under combinations of different confining stresses and stress difference ratios were compared with the predictions of the intelligent displacement inversion algorithm.

### 3.1 Acquisition of calculated parameters

The model parameters of Shanghai soft clay are listed in Table 2. These parameters are based on the research of Li [38] and Wei [39].

**Table 2:** Model parameters of Shanghai soft clay [38].

*λ*	*κ*	*M*	*v*	*e*_0_	*C*_*a*_	*R*	*ζ*
0.20	0.041	1.49	0.3	1.45	0.0172	2.0	10.0

Some parameters of Shanghai soft clay, such as the shape parameter *R* and constant ζ, are derived from Li’s elasto-viscoplastic constitutive model. The physical properties of the soft clay used in this study include *λ* = 0.20, *κ* = 0.041, *e*_0_ = 1.45, *and φ*_*r*_ = 32°. The residual internal friction angle was obtained by numerical calculations. In addition, the fractional differential model parameters *E*_1_, *E*_2_, *η*_1_, *q*_1_, *η*_2_ *and q*_2_ were identified using the intelligent displacement inversion algorithm developed in this study. Based on the variation in the soft clay properties, the ranges of the five parameters to be inverted can be determined as follows (the parameter levels are listed in Table 3):

- Elastic modulus *E*_2_ of the viscoelastic body: 1.0–10.0 MPa;
- Viscosity coefficient *η*_1_ of the viscoelastic body: 100.0–1000.0 *GPa*⋅*min*;
- Viscosity coefficient *η*_2_ of the viscoplastic body: 100.0–1000.0 *GPa*⋅*min*;
- Differential order *q*_1_ of the fractional differential element of the viscoelastic body: 0.1–1.0;
- Differential order *q*_2_ of the fractional differential element of the viscoplastic body: 0.1–1.0.

**Table 3:** Parameter levels of the fractional differential constitutive model of soft clay.

level	*E*_2_(MPa)	*η*_1_(*GPa*⋅*min*)	*η*_2_(*GPa*⋅*min*)	*q*_1_	*q*_2_
1	1.0	100.0	100.0	0.1	0.1
2	3.0	300.0	400.0	0.3	0.3
3	5.0	500.0	600.0	0.5	0.5
4	8.0	800.0	800.0	0.7	0.7
5	10.0	1000.0	1000.0	1.0	1.0

Based on the principle of the orthogonal design, 25 test groups were developed, as shown in Table 4. For each test group, the developed fractional differential constitutive model of soft clay was used to calculate the axial strain values for the five rheological periods. Altogether, a learning sample database that includes 125 data samples was established. Statistical analysis of test groups is shown in Table 5.

**Table 4:** Details of test groups used in this study.

Group	Parameters to be inverted					Axial strain value (%) of each period (H)				
	*E*_2_	*η*_1_	*η*_2_	*q*_1_	*q*_2_	20	40	60	80	100
1	1.0	100.0	100.0	0.1	0.1	2.2252	2.3175	2.3715	2.4094	2.4383
2	1.0	300.0	400.0	0.3	0.3	0.9793	1.1614	1.2792	1.3678	1.4394
3	1.0	500.0	600.0	0.5	0.5	0.2913	0.4049	0.4894	0.5590	0.6190
4	1.0	800.0	800.0	0.7	0.7	0.0634	0.1025	0.1356	0.1653	0.1926
5	1.0	1000.0	1000.0	1.0	1.0	0.0068	0.0137	0.0205	0.0273	0.0341
6	3.0	100.0	400.0	0.5	0.7	0.3350	0.4458	0.5221	0.5812	0.6297
7	3.0	300.0	600.0	0.7	1.0	0.0887	0.1414	0.1848	0.2228	0.2570
8	3.0	500.0	800.0	1.0	0.1	0.0132	0.0262	0.0392	0.0520	0.0648
9	3.0	800.0	1000.0	0.1	0.3	0.6987	0.7290	0.7469	0.7597	0.7696
10	3.0	1000.0	100.0	0.3	0.5	0.3180	0.3776	0.4161	0.4452	0.4687
11	5.0	100.0	600.0	1.0	0.3	0.0641	0.1244	0.1812	0.2347	0.2851
12	5.0	300.0	800.0	0.1	0.5	0.4587	0.4771	0.4877	0.4949	0.5001
13	5.0	500.0	1000.0	0.3	0.7	0.2559	0.3000	0.3280	0.3488	0.3654
14	5.0	800.0	100.0	0.5	1.0	0.1022	0.1418	0.1713	0.1956	0.2165
15	5.0	1000.0	400.0	0.7	0.1	0.0330	0.0530	0.0696	0.0844	0.0978
16	8.0	100.0	800.0	0.3	1.0	0.2512	0.2866	0.3082	0.3237	0.3359
17	8.0	300.0	1000.0	0.5	0.1	0.1194	0.1594	0.1871	0.2086	0.2263
18	8.0	500.0	100.0	0.7	0.3	0.0455	0.0718	0.0932	0.1116	0.1281
19	8.0	800.0	400.0	1.0	0.5	0.0082	0.0163	0.0244	0.0323	0.0401
20	8.0	1000.0	600.0	0.1	0.7	0.2745	0.2861	0.2929	0.2977	0.3014
21	10.0	100.0	1000.0	0.7	0.5	0.1171	0.1735	0.2139	0.2452	0.2707
22	10.0	300.0	100.0	1.0	0.7	0.0220	0.0429	0.0631	0.0824	0.1010
23	10.0	500.0	400.0	0.1	1.0	0.2324	0.2422	0.2480	0.2521	0.2551
24	10.0	800.0	600.0	0.3	0.1	0.1348	0.1576	0.1720	0.1826	0.1911
25	10.0	1000.0	800.0	0.5	0.3	0.0619	0.0843	0.1005	0.1134	0.1242

**Table 5:** Statistical analysis of test groups.

Statistical Analysis	Minimum	Maximum	Mean	Standard Deviation	Range
***E***_**2**_	1.0	10.0	5.4	3.2	1.0–10.0
***η***_**1**_	100.0	1000.0	540.0	326.2	100.0~1000.0
***η***_**2**_	100.0	1000.0	580.0	312.4	100.0~1000.0
***q***_**1**_	0.1	1.0	0.5	0.3	0.1 ~1.0
***q***_**2**_	0.1	1.0	0.5	0.3	0.1 ~1.0
**Axial Strain(%)**	2.4383	0.0068	0.3587	0.4879	/

Optimization of the parameters of the LSSVM algorithm was done as follows: The population size of the particle swarm was set as 50, and the particle dimension was set as 2, which were the model regularization parameter *C* and kernel parameter *δ*, respectively. The maximum evolution algebra, initial inertia weight, and final inertia weights were 1000, 0.95, and 0.2, respectively. The maximum and minimum values of *c*_1_, *c*_2_ were 2 and 0.2, respectively. The determination distance of the dynamic neighborhood was defined as *Frac* = (3*t+0.6*N)/*N*, where *t* represented the number of iterative steps, and *N* represented the total number of iterative steps. The selection of the dynamic neighborhood of a particle was based on the fact that the ratio of *dis*_*ij*_ to *dis*_*max* should be greater than the critical determination distance *Frac* [36].

K-fold cross validation divided the sample set into k subsets, any combination of k-1 subsets forms a learning set, and the remaining one was used as a test set for training. The training was repeated k times so that all samples could participate in the training and tests. In this way, the final parameters could satisfy the requirement of the minimizing sum of squares of the predicted errors. In this section, set the k value as 5. The first 100 samples in the learning sample database were used as learning samples, and the last 25 were used as test samples. At the same time, the fitness function was defined as the least mean square error of the test sample prediction. The search range of the model regularization parameter *C* and kernel parameter *δ* was set as 0.01 to 500, respectively. After optimization by the SADPSO algorithm, the LSSVM model parameters were further optimized by 3-fold cross validation.

The establishment of the LSSVM model was done as follows: the LSSVM model of the fractional differential constitutive model was established using the above parameters. Its learning sample set was composed of 5 axial strain values of the rheological period under each group of test results.

Using the SADPSO algorithm to invert parameters according to the measured displacement, the population size of the particle swarm was set to 50, and the particle dimension was set to 5, i.e., *E*_2_, *η*_1_, *q*_1_, *η*_2_ *and q*_2_. The maximum evolution algebra, initial inertia weight, final inertia weight, and other parameters were set as mentioned above. From the learning sample database, 125 samples were used as the learning sample set, and the model parameter values represented by each particle and axial strain values of different rheological segments were used as the test sample set. The fitness function was defined to minimize the mean square error between the predicted axial deformation value of the test sample set in the LSSVM model and the actual axial deformation value.

Finally, the parameters of the fractional differential constitutive model are as follows: *E*_2_ = 6.05 *MPa*; *η*_1_ = 100 G*Pa*⋅*min*; *q*_1_ = 0.304; *η*_2_ = 200.33 *GPa*⋅*min; q*_2_ = 0.303.

### 3.2 Validation of the model

**Fig 4:**
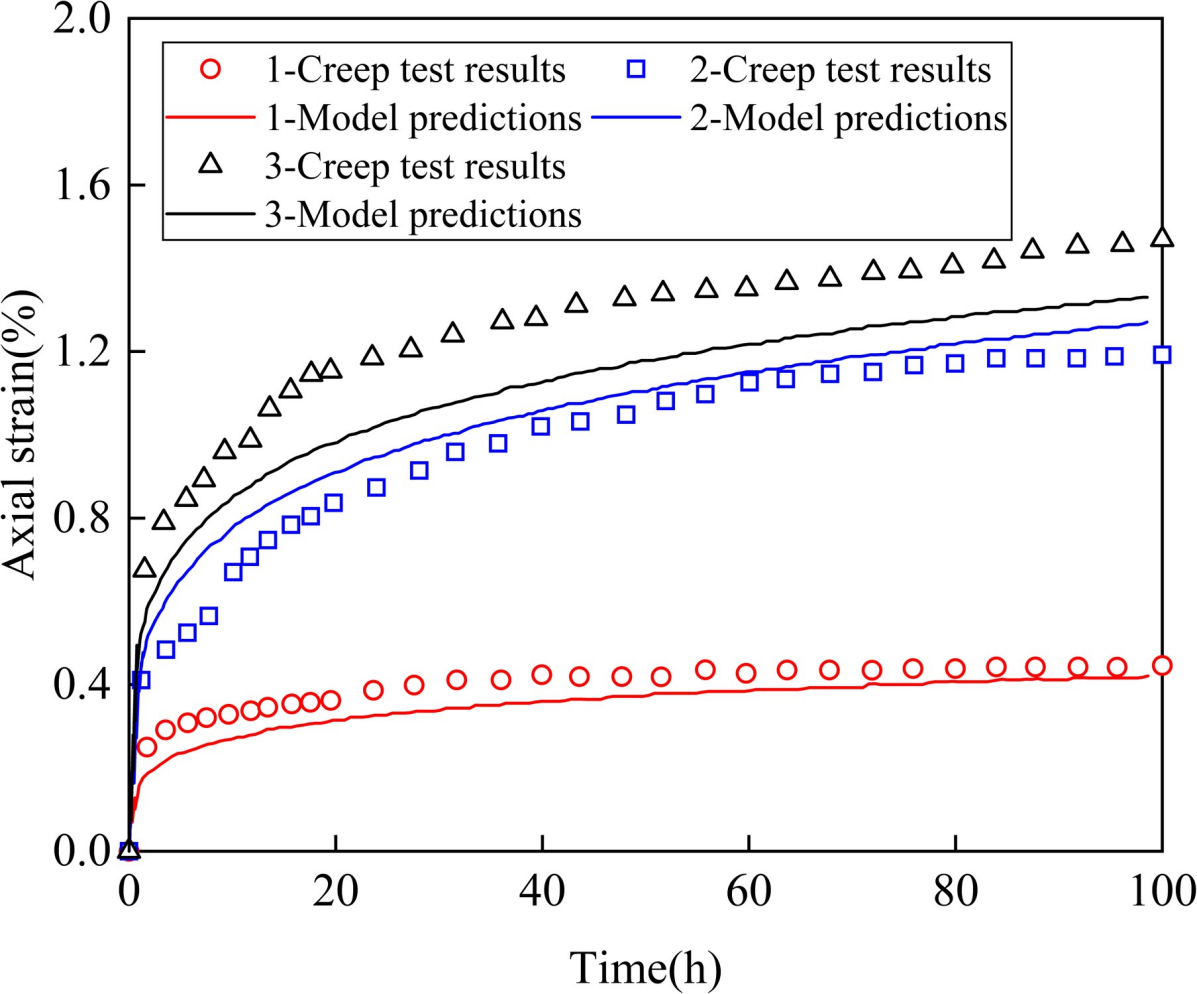
Comparison curve between the fractional differential constitutive model and undrained triaxial creep test (Condition 1, confining stress: 100 kPa, stress difference ratio: 0.25; Condition 2, confining stress: 100 kPa, stress difference ratio: 0.50; Condition 3, confining stress: 200 kPa, stress difference ratio: 0.50).

The test results were fitted to obtain the parameters required for the model calculation. Then, the axial strain values were calculated and compared with the experimental results. Finally, the reliability of the model is validated under different conditions as follows:

- Condition 1: Based on the model parameters obtained by back analysis, the axial creep deformation of soft clay in East China was calculated under a 100 kPa confining pressure and deviator stress ratio of d = 0.25. A comparison of the calculation results is shown in Fig 4.
- Condition 2: When the confining pressure was 100 kPa and the deviator stress ratio was d = 0.5, the axial creep deformation value of soft clay in East China was calculated and compared with the experimental value of axial creep deformation. A comparison of the results is shown in Fig 4.
- Condition 3: When *p*_*c*_ = 200 *kPa* and other model parameter values were the same as above, the axial creep deformation value of soft clay in East China was calculated under the action of a 200 kPa confining pressure and a deviator stress ratio d = 0.5. The comparison results are shown in Fig 4.

It can be seen from Fig 4 that the values calculated in this study are similar to those of the test results. The coefficient of determination (R^2^) and mean absolute error (MAE) for three conditions are: 0.96 and 0.04, 0.94 and 0.06, 0.79 and 0.13. The results show that the fractional differential constitutive model for soil clay and the intelligent displacement inversion algorithm defined in this study are reasonable and effective.

There are some deviations between the calculated and test results. The proposed model is established on the assumption that 11 parameters are constant in the same soil layer along the depth of the soil layer. This assumption means that the stress-strain relationship of the soft clay is based on the premise that it does not change with the depth at the same soil layer. However, due to natural settlement, the self-weight stress distributed along the depth already exists in the soil layer. The nonlinear stress-strain relationship at different soil layer depths should be different.

## 4. Engineering application

As an integrated part of the transportation infrastructure, the expressways need the assessing of the stability and settlement during construction. To demonstrate the engineering application value of the fractional differential constitutive model proposed in this study, the stability of the subgrade structure and post-construction settlement were calculated.

Based on the Expressway Project in South China, the constructor adopted an overload preloading foundation with a plastic drainage board scheme. The deformation of the soft soil foundation was calculated on a road section. The road section had a thick soil layer (approximately 10.70–33.70 m) and a high subgrade filling (the height is 4.1–5.3 m). Using static sounding, the distribution range and thickness of the soft soil were further detected, which provided basic data for the analysis and evaluation of the reinforcement effect.

The theoretical model was applied using the FEM, and the calculation results were compared with the actual results. Some soil parameters used in the modeling can be accurately obtained using the fractional differential constitutive model, and the process is similar to the above test. This theory can help the designer in calculating the soft soil foundation settlement and the engineering manager in determining the optimal foundation reinforcement scheme.

### 4.1 Calculation overview

The parameters were simplified to facilitate engineering calculations. The calculated depth of the soft foundation was 22.5 m, the calculated width was 50 m, the top width of the embankment was 2 × 16 m, and the bottom width of the embankment was 2 × 23.5 m.

The soil layer included a muddy clay layer with a thickness of 13 m, muddy sub-clay layer with a thickness of 3 m, and silty clay layer with a thickness of 6.5 m. The filling soil height was 10.9 m, and the duration was 575 days.

### 4.2 Stratigraphic data

The road section included four layers: muddy clay, muddy sub-clay, silty clay, and filling soil. Each soil layer includes instantaneous elastic parameters *E*_1_, viscoelastic parameters *E*_2_, *η*_1_, *q*_1_, plastic parameters *λ*, *κ*, *e*_0_, *φ*_*r*_, *p*_*c*_, viscoplastic parameters *η*_2_, *q*_2_, and Poisson ratio *μ*. There are a total of 12 parameters, among which the plastic parameters and Poisson’s ratio were obtained by experimental methods, as shown in Table 6, and the other parameters were calculated by the intelligent displacement inversion method proposed in this study.

**Table 6:** Parts of the experimental parameters of the fractional differential constitutive model.

Soil layer	*μ*	*λ*	*κ*	*e*_0_	*φ*_*r*_(°)	*p*_*c*_ (kPa)	*γ* (kN/m^3^)	Permeability Coefficient K(m/d)
Muddy clay	0.36	0.242	0.02	1.70	18.0	110	17	2.03×10^−4^
Muddy clay	0.34	0.402	0.041	1.76	13.4	244.9	15.9	2.03×10^−4^
Silty clay	0.30	0.375	0.03	1.69	15.5	321.68	16.3	9.59×10^−5^
Filling soil	0.30	0.06	0.005	0.668	27.1	31.5	21	1

Due to the limited engineering data, *λ*, *κ*, *φ*_*r*_ and Poisson ratio *μ* were obtained from the literature [40], and *e*_0_ and *p*_*c*_ were obtained by the method of engineering estimation. Therefore, 24 model parameters were recognized using the intelligent displacement inversion method.

The intelligent displacement inversion method was used to inversely calculate the value of fractional differential model parameters for each layer of the soil material. The ranges of the six parameters to be inverted were determined as follows:

- Instantaneous elastic modulus *E*_1_ of the viscoelastic body: 1.0–100.0 MPa;
- Elastic modulus *E*_2_ of the viscoelastic body: 1.0–100.0 MPa;
- Viscosity coefficient *η*_1_ of the viscoelastic body: 1.0–1000.0 *MPa*⋅*day*;
- Viscosity coefficient *η*_2_ of the viscoplastic body: 1.0–1000.0 *MPa*⋅*day*;
- Differential order *q*_1_ of the fractional differential element of the viscoelastic body: 0.1–1.0;
- Differential order *q*_2_ of the fractional differential element of the viscoplastic body: 0.1–1.0.

This section takes five value levels for each parameter, and the specific corresponding values of the value levels are shown in Table 7.

**Table 7:** Parameter level of the fractional differential constitutive model on soft soil.

Value levels	*E*_1_ (MPa)	*E*_2_ (MPa)	*η*_1_ (*MPa*⋅*day*)	*η*_2_ (*MPa*⋅*day*)	*q*_1_	*q*_2_
1	1.0	1.0	1.0	1.0	0.1	0.1
2	30.0	40.0	300.0	400.0	0.3	0.3
3	50.0	60.0	500.0	600.0	0.5	0.5
4	80.0	80.0	800.0	800.0	0.7	0.7
5	100.0	100.0	1000.0	1000.0	1.0	1.0

The road base was selected as the finite-element calculation point. A learning sample library was constructed based on the principle of orthogonal design. The fractional differential constitutive model parameters *E*_1_, *E*_2_, *η*_1_, *q*_1_, *η*_2_, *q*_2_ and rheological time *t* were used as the input variables of the learning sample. The finite element calculation value corresponding to the calculation point of time *t* was used as the output variable. The project included 4 soil layers, each of which had 25 sets of test results. For each test group, the developed fractional differential constitutive model of soft clay was used to calculate the axial strain values of the rheological periods. Altogether, a learning database that included 1000 data samples was established.

Optimization of the parameters of LSSVM algorithm was done as follows: the first 750 samples in the learning sample database were used as learning samples, and the last 250 samples were used as test samples. At the same time, the fitness function was defined as the least mean square error of the test sample prediction. The search range of the model regularization parameter *C* and kernel parameter *δ* were set as 0.01 to 500. After optimization using the SADPSO algorithm, the LSSVM model parameters were further optimized by 3-fold cross validation.

Establishment of the LSSVM model was done as follows: the LSSVM model of the fractional differential constitutive model was established using the above parameters. The learning sample set was composed of 1000 sample data. As before, the project included 4 soil layers, and each soil layer contained 25 sets of test results. For each test group, the developed fractional differential constitutive model of soft clay was used to calculate the axial strain values of ten rheological periods. Therefore, a learning database including 1000 sample data is established.

The SADPSO algorithm was used to invert the parameters according to the measured displacement, maximum evolution algebra, initial inertia weight, final inertia weight, and other parameters were set as above. 1000 samples in the learning sample database were used as the learning sample set. The model parameter values represented by each particle and the actual settlement value under different rheological sections were used as the test sample set. The fitness function was defined to minimize the mean square error between the predicted settlement value of the test sample set in the LSSVM model and the actual settlement value.

The parameters of the fractional differential constitutive model are presented in Table 8.

**Table 8:** Parts of back analytical parameters of the fractional differential constitutive model.

Soil layer	*E*_1_ (MPa)	*E*_2_ (MPa)	*η*_1_ (*MPa*⋅*day*)	*η*_2_ (*MPa*⋅*day*)	*q*_1_	*q*_2_
Muddy clay	2.7	10.2	33.5	95.0	0.30	0.30
Muddy sub-clay	5.1	14.5	26.0	80.0	0.30	0.30
Silty clay	7.5	18.5	23.0	60.0	0.31	0.31
Filling soil	10.0	29.5	20.0	55.5	0.30	0.30

### 4.3 Settlement value

**Fig 5:**
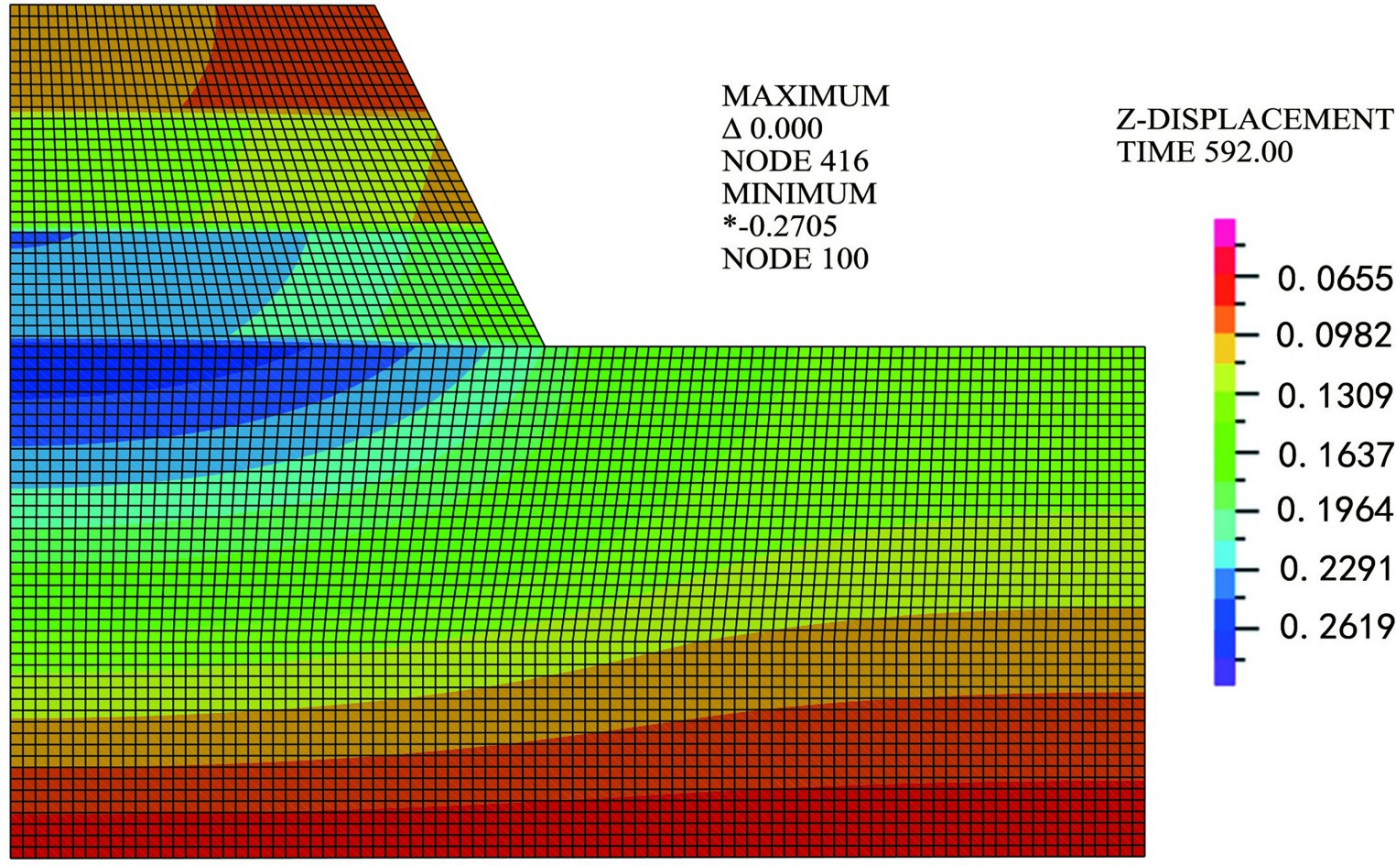
Vertical displacement on the 592^nd^ day.

In this section, the finite element model of the soft soil foundation settlement is established using finite element software, and the meshing diagram of the model is shown in Fig 5.

The model size and soil layer were the same as those in the simplified road section above. The design size of the calculation model is as follows: the calculation depth of the soft foundation is 22.5 m, the calculation width is 50 m, the top width of the embankment is 2 × 16 m, and the bottom width of the embankment is 2 × 23.5 m. The soil layer includes a silty clay layer with a thickness of 13 m; a silty loam layer with a thickness of 3 m; a loam layer with a thickness of 6.5 m; a filling height of 10.9 m; and the total duration was 575 days.

**Fig 6:**
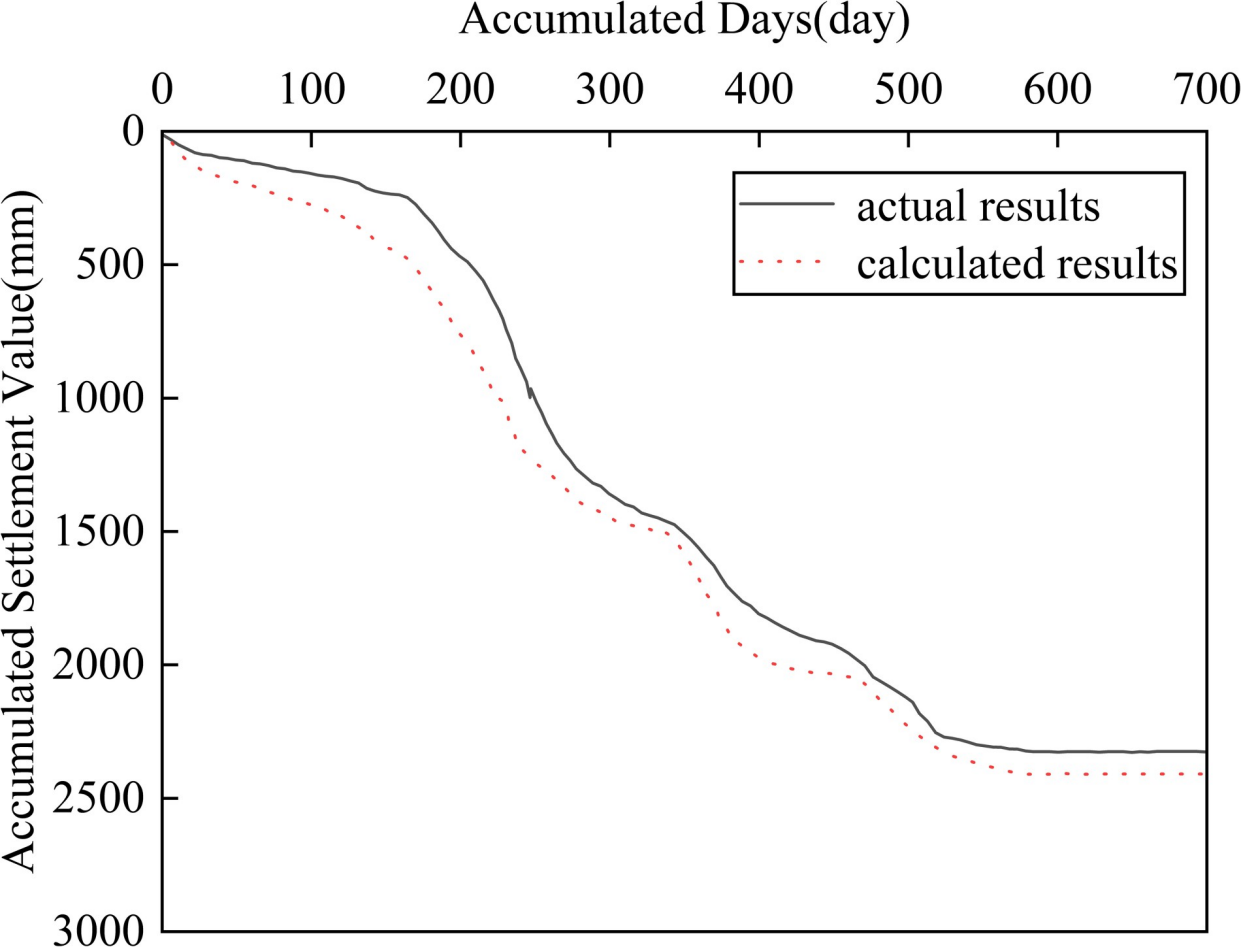
Settlement-time curve of the center of the subgrade surface.

**Fig 7:**
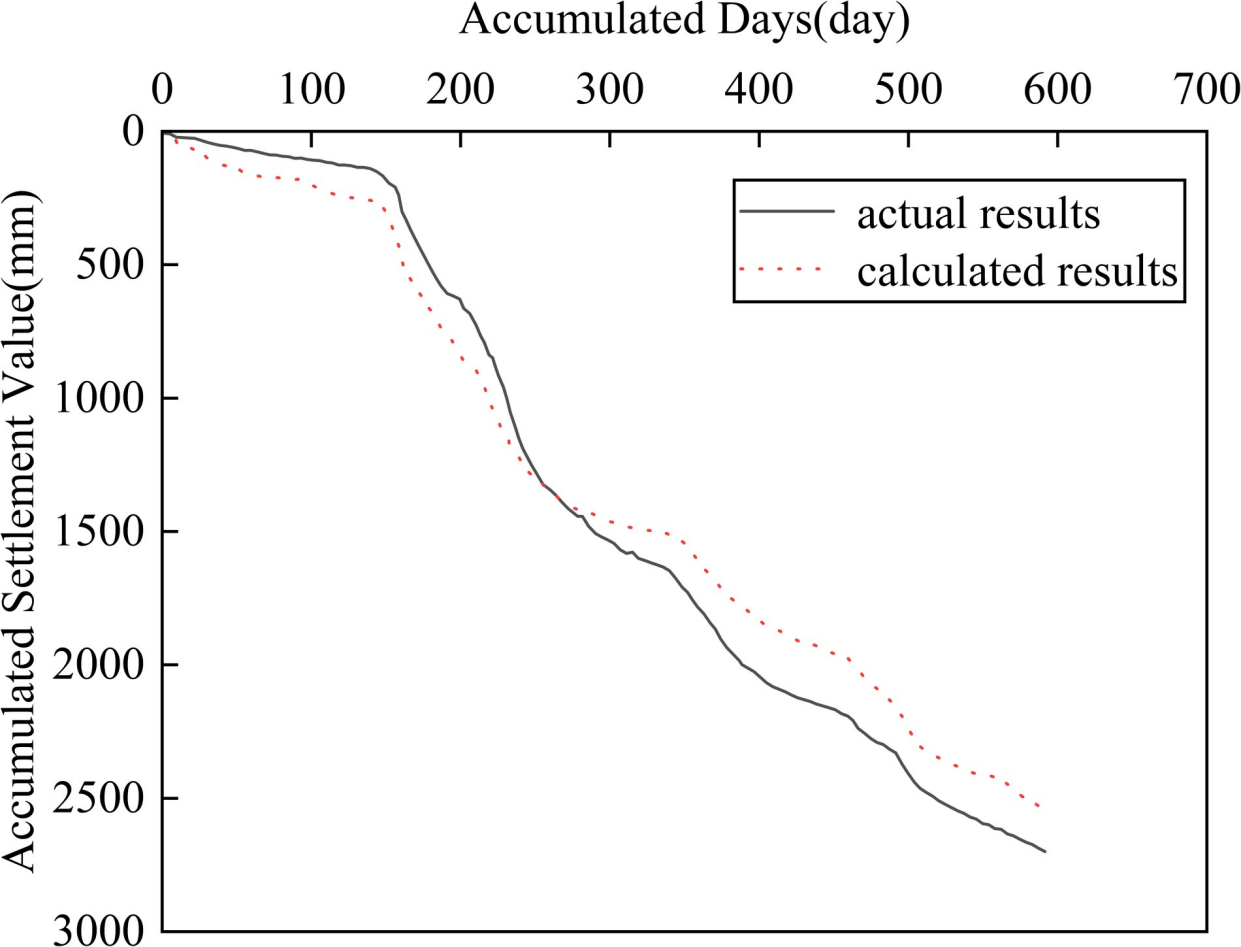
Settlement-time curve of the 1.5 m height.

The calculation results of the FEM are shown in Figs 5–7.

1. Fig 5 shows a cloud diagram of the vertical deformation distribution of the soft soil foundation when the loading is completed. As shown in Fig 5, the maximum vertical deformation occurred at the center of the road base surface, and the maximum vertical deformation was 2705 mm.
2. Fig 6 shows the settlement-time curve at the center of the road base surface and Fig 7 shows the settlement-time curve at a 1.5 m height. As shown in Figs 6 and 7, the calculation results of the fractional differential constitutive model can better follow the rules of the actual soil settlement. Many factors influencing soft foundations have been considered, such as the consolidation settlement of the soil and rheology of the soil [41]. Therefore, the fractional differential constitutive model was validated for accuracy.

As shown in Figs 6 and 7, the settlement results correlate with the actual settlement of the soil, which demonstrates the validity and rationality of the fractional differential constitutive model used in this study. However, there are some deviations between the calculated and actual monitoring results. The main reasons are:

1. Owing to the complicated engineering environment, the simplified calculation model could have caused the finite deviations in the calculation results.
2. The deformation of a soft soil foundation is a developing process. However, the fractional differential constitutive model describes limited properties of the soil, such as nonlinear rheology and primary-secondary consolidation coupling deformation [42]. This limitation of the model causes the calculation results to deviate.
3. The influence of the parameters of the fractional differential constitutive model, including the plastic parameters and Poisson’s ratio obtained by the experimental method, and the intelligent inversion parameters obtained from the model. Different parameter sources can lead to different deviations.

## 5. Conclusion

In this study, a fractional differential constitutive model of soft clay was developed based on the coupling of primary and secondary consolidations of soft clay. Soft clay’s strain consists of four aspects: instantaneous elastic, instantaneous plastic, viscoelastic, and viscoplastic, so it could better show the complex characteristics of soft clay such as the nonlinear rheology.

This paper proposed an intelligent displacement inversion algorithm for improving the modeling process’s efficiency, which was the statistical adaptive dynamic neighborhood particle swarm optimization. This algorithm could solve problems like changing inertia weight and optimizing functions.

The finite element results showed that the improved algorithm could better reflect the objective law of soil settlement. The calculation of parameters was close to the actual data, and the calculation errors were within the acceptable range of engineering.

Overall, the fractional differential constitutive model and intelligent displacement inversion algorithm were reasonable and practical in this paper.
